# Supplementary material for: An Exercise Immune Fitness Test to Unravel Disease Mechanisms—A Proof-of-Concept Heart Failure Study
Source: J Clin Med. 2024 May 29;13(11):3200. doi: 10.3390/jcm13113200 (PMC11172881; doi:10.3390/jcm13113200)
Supplement: Supplementary file 1 [file jcm-13-03200-s001.zip › jcm-3035081-supplementary.pdf]

Table S1: Gene ontology information on the 11 genes identified as described in the text.

Gene ID Symbol	Summary
100287898 *TTC34*	TTC34 (Tetratricopeptide Repeat Domain 34) is a Protein Coding gene. A link with systemic lupus erythematosus is proposed [37].
100873932 *DPYD-AS1*	DPYD-AS1 (DPYD Antisense RNA 1) is a non-coding RNA Gene. DPYD is a complex gene whose expression is epigenetically regulated by long non-coding RNAs (lncRNAs) within the locus, modules inflammatory pathways [38] and is potentially linked to Severe Takotsubo Cardiomyopathy [39].
57821 *CCDC181*	CCDC181 (Coiled-Coil Domain Containing 181) is a Protein Coding gene. Diseases associated with CCDC181 include astrocytoma, chordoid glioma and childhood brain stem glioma. Ccdc181 seems to interact directly with microtubules and localizes to the microtubular manchette of elongating spermatids; reported as a putative prognostic factor for prostate cancer [40].
338773 *TMEM119*	TMEM119 (Transmembrane Protein 119) is a Protein Coding gene. Among its related pathways are Microglia Activation During Neuroinflammation, bone formation and normal bone mineralization, and promotion of the differentiation of myoblasts into osteoblasts [41].
51330 *TNFRSF12A*	TNFRSF12A (TNF Receptor Superfamily Member 12A) is a Protein Coding gene. Among its related pathways are Cytokine Signaling in the Immune system and CDK-mediated phosphorylation and removal of Cdc6. It is a weak inducer of apoptosis in some cell types. Promotes angiogenesis and the proliferation of endothelial cells. May modulate cellular adhesion to matrix proteins. Tnfrsf12a is highly inducible and plays a key role in the development of cardiac hypertrophy and heart failure [42]. TNFRSF12A, which is a cell surface-associated type II transmembrane protein, has multiple biological activities, including stimulation of cell growth and angiogenesis, induction of inflammatory cytokines, and under some experimental conditions, stimulation of apoptosis [43].
64073 *C19orf33*	C19orf33 (Chromosome 19 Open Reading Frame 33) is a Protein Coding gene. Diseases associated with C19orf33 include Eclampsia and Pre-Eclampsia. Gene Ontology (GO) annotations related to this gene include double-stranded DNA binding and single-stranded DNA binding. The protein encoded by this gene has been shown to be upregulated in SV40-immortalized fibroblasts as well as in endometrial carcinoma cells. This protein may play a role in placental development and diseases such as pre-eclampsia. Two transcript variants encoding different isoforms have been found for this gene. C19orf33 expression in several cancers, including pancreatic cancer, and is closely related to the patient's prognosis. C19orf33 inhibits breast cancer and papillary thyroid carcinoma progression by regulating EMT or YAP1 coordination in the Hippo pathway [44], respectively C19orf33 was found to be differentially expressed (downregulated) regarding ischemic cardiomyopathy (ICM) compared to normal controls [45].

3397<br>*ID1*	ID1 (Inhibitor of DNA Binding 1, HLH Protein) is a Protein Coding gene. Among its related pathways are the ID signaling pathway and the Hippo signaling pathway. Gene Ontology (GO) annotations related to this gene include DNA-binding transcription factor activity and protein dimerization activity. The protein encoded by this gene is a helix–loop–helix (HLH) protein that can form heterodimers with members of the basic HLH family of transcription factors. The encoded protein has no DNA binding activity and therefore can inhibit the DNA binding and transcriptional activation ability of basic HLH proteins with which it interacts. This protein may play a role in cell growth, senescence, and differentiation and is implicated in regulating a variety of cellular processes, including cellular growth, senescence, differentiation, apoptosis, angiogenesis, and neoplastic transformation. Inhibits skeletal muscle and cardiac myocyte differentiation. Regulates the circadian clock by repressing the transcriptional activator activity of the CLOCK-ARNTL/BMAL1 heterodimer. Recent studies have demonstrated that Ids (Inhibitors of DNA binding genes) are related to multiple aspects of cardiovascular diseases, including congenital structural, coronary heart disease, and arrhythmia, severe cardiac defects including valvular and septal defects, outflow tract atresia, impaired ventricular trabeculation and thinning of the compact myocardium layers. Functionally, the expression of Id1 induces apoptosis in cardiac myocytes.
84076<br>*TKTL2*	TKTL2 is a Protein Coding Gene. Gene Ontology (GO) annotations related to this gene include oxidoreductase activity, acting on the aldehyde or oxo group of donors, disulfide as acceptor and transketolase activity. An important paralog of this gene is TKTL1. Predicted to enable thiamine pyrophosphate binding activity and transketolase activity. Located in cytoplasm. Plays an essential role in total transketolase activity and cell proliferation in cancer cells; after transfection with anti-TKTL1 siRNA, total transketolase activity dramatically decreased and proliferation was significantly inhibited in cancer cells. TKTL2 was expressed in varying numbers of CC (colon cancer) tissues compared to no measurable expressions in all NC (normal colon) tissue specimens, making it a suitable candidate for CC marker **[**46**].**
221981<br>*THSD7A*	THSD7A (Thrombospondin Type 1 Domain Containing 7A) is a Protein Coding gene. Diseases associated with THSD7A include Membranous Nephropathy and Bronchiectasis 3. Among its related pathways are Diseases associated with O-glycosylation of proteins and Metabolism of proteins. The protein encoded by this gene is found almost exclusively in endothelial cells from the placenta and umbilical cord. The encoded protein appears to interact with alpha(v)beta (3) integrin and paxillin to inhibit endothelial cell migration and tube formation. This protein may be involved in cytoskeletal organization. Variations in this gene may be associated with low bone mineral density in osteoporosis. THSD7A was found to be a gene associated with the promotion of atherosclerosis and coronary artery disease (CAD) **[**47**].**
100131439<br>*CD300LD*	CD300LD (CD300 Molecule Like Family Member D) is a Protein Coding gene. Diseases associated with CD300LD include Ectodermal Dysplasia 11A, Hypohidrotic/Hair/Tooth Type, and Autosomal Dominant. Among its related pathways are the Innate Immune System and Class I MHC-mediated antigen processing and presentation. Predicted to enable transmembrane signaling receptor activity and virus receptor activity. Predicted to be involved in the immune system process. CD300LD is predicted to act upstream of or within the regulation of interleukin-6 production and regulation of tumor necrosis factor production and correlates with metabolic disease, inflammatory response, organismal injury, and abnormalities according to Ingenuity Pathway Analysis **[**48**].**

146664 *MGAT5B*	MGAT5B (Alpha-1,6-Mannosylglycoprotein 6-BetaNAcetylglucosaminyltransferase B) is a Protein Coding gene. Diseases associated with MGAT5B include Walker–Warburg Syndrome, which is predicted to be an integral component of the membrane and active in the Golgi apparatus. MGAT5B was found to be a Transcriptomic Diagnostic Biomarker for Detection of Patients with Myocarditis [49].

Table S2: Gene ontology information on the six overlapping genes.

**Gene ID/Symbol**	**Summary**
90853 *SPOCD1*	The SPOCD1 gene, also known as "SPOC domain containing 1", is a gene found in humans. It encodes a protein that contains a SPOC (Spen paralog and ortholog C-terminal) domain. SPOC domains are involved in various cellular processes, including transcriptional regulation and RNA processing. The specific function of the SPOCD1 gene and its protein product are not yet fully understood, and research is ongoing to elucidate its roles in cellular physiology and potential implications in health and disease. This gene has been found to accelerate progression and metastasis in colorectal cancer[50], ovarian cancer [51], and esophageal cancer [52].
200958 *MUC20*	The MUC20 gene encodes a protein known as Mucin 20, which is a member of the mucin protein family. Mucins are primarily found on the surface of epithelial cells, where they form a protective barrier. They play important roles in lubricating and protecting various mucosal surfaces in the body, such as the gastrointestinal tract, respiratory tract, and reproductive tract. MUC20 overexpression has been shown to significantly decrease esophageal cancer cell viability [53] and also in a number of other gastrointestinal cancers. The presence of this gene has also been proven to be a prognostic biomarker for clear cell renal cell carcinoma. Its presence was positively correlated with survival and negatively correlated with clinicopathologic characteristics [54].
10439 *OLFM1*	Olfactomedin-1 (OLFM1) is expressed in various tissues, including the brain, retina, and testis, and it plays important roles in neural development, axon guidance, and cell adhesion. OLFM1 may have implications for certain diseases and conditions such as depression and mania [55], defective olfaction and impaired female fertility [56].
773 *CACNA1A*	The CACNA1A gene encodes the alpha -1A subunit of the P/Q type voltage-dependent calcium channel. These channels are crucial for neurotransmitter release at synapses and for regulating neuronal excitability. Mutations in the CACNA1A gene have been associated with various neurological disorders, including episodic ataxia type 2, familial hemiplegic migraine type 1, and spinocerebellar ataxia type 6 [57]. Mutations in the gene have also been found to induce epilepsy [58]
8653 *DDX3Y*	The DDX3Y gene is located on the Y chromosome and encodes a protein belonging to the DEAD-box RNA helicase family. It plays an essential role in RNA metabolism, including transcription, translation, and decay [59]. Its overexpression was also found to play a role in the development of human non-obstructive azoospermia [60] which renders males infertile. Some research also suggests that DDX3Y may also play an important role in innate immunity by interacting with viral RNA during viral infections.
286554 BCORP1	BCORP1 is a pseudogene involved in increased transferrin endocytosis.
